# Effect of Pictorial Depth Cues, Binocular Disparity Cues and Motion Parallax Depth Cues on Lightness Perception in Three-Dimensional Virtual Scenes

**DOI:** 10.1371/journal.pone.0003177

**Published:** 2008-09-10

**Authors:** Michiteru Kitazaki, Hisashi Kobiki, Laurence T. Maloney

**Affiliations:** 1 Research Center for Future Vehicle, Toyohashi University of Technology, Tempakucho, Aichi, Japan; 2 Corporate Research & Development Center, Toshiba Corporation, Saiwaiku, Kanagawa, Japan; 3 Department of Psychology, and also Center for Neural Science, New York University, New York, New York, United States of America; James Cook University, Australia

## Abstract

**Background:**

Surface lightness perception is affected by scene interpretation. There is some experimental evidence that perceived lightness under bi-ocular viewing conditions is different from perceived lightness in actual scenes but there are also reports that viewing conditions have little or no effect on perceived color. We investigated how mixes of depth cues affect perception of lightness in three-dimensional rendered scenes containing strong gradients of illumination in depth.

**Methodology/Principal Findings:**

Observers viewed a virtual room (4 m width×5 m height×17.5 m depth) with checkerboard walls and floor. In four conditions, the room was presented with or without binocular disparity (BD) depth cues and with or without motion parallax (MP) depth cues. In all conditions, observers were asked to adjust the luminance of a comparison surface to match the lightness of test surfaces placed at seven different depths (8.5–17.5 m) in the scene. We estimated lightness versus depth profiles in all four depth cue conditions. Even when observers had only pictorial depth cues (no MP, no BD), they partially but significantly discounted the illumination gradient in judging lightness. Adding either MP or BD led to significantly greater discounting and both cues together produced the greatest discounting. The effects of MP and BD were approximately additive. BD had greater influence at near distances than far.

**Conclusions/Significance:**

These results suggest the surface lightness perception is modulated by three-dimensional perception/interpretation using pictorial, binocular-disparity, and motion-parallax cues additively. We propose a two-stage (2D and 3D) processing model for lightness perception.

## Introduction

Much previous research concerning lightness perception makes use of stimuli that are effectively pictures of scenes, but viewed with both eyes. With scenes viewed “bi-ocularly” in this way, there is potential conflict between pictorial cues to depth and depth cues such as binocular disparity and motion parallax that are consistent with the flat surface of the picture viewed (the term “bi-ocular” refers to viewing conditions where the observer views a picture (a two-dimensional projection) of a three-dimensional scene with both eyes [1]). There is some experimental evidence that perceived lightness under bi-ocular viewing conditions is different from perceived lightness in actual scenes (e.g. [2]) but there are also reports that viewing conditions have little or no effect on perceived color [3].

In this paper we first describe why the depth interpretation of a scene should affect surface lightness perception when the flow of light in the scene is not uniform. Next we review the literature concerning lightness perception in three-dimensional scenes and examine what role specific depth cues play in experimental design. We then report an experiment contrasting bi-ocular perception of three-dimensional scenes with viewing of identical scenes with binocular disparity and/or motion parallax cues to depth also available. The scenes all had strong gradients of illumination in depth. To anticipate our conclusion, we find that added depth cues markedly alter lightness perception and lead to an increased degree of lightness constancy.

### The light field

**Figure 1:**
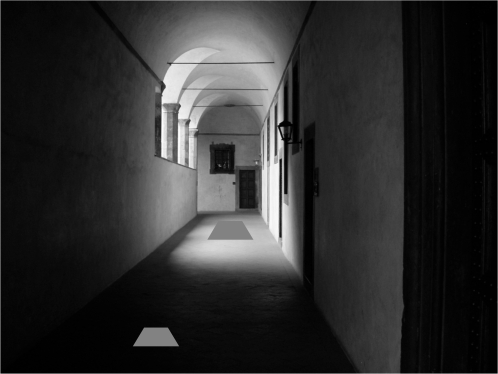
An everyday scene. We superimposed two trapezoidal patches that are identical in albedo and size on the picture. Interpreted as part of the picture, they differ markedly in apparent albedo and apparent size.

If we could insert a neutral matte surface patch at different locations in the scene pictured in Figure 1, the intensity of light emitted by the patch would vary with the location and orientation of the patch with respect to the sources of light in the scene. The light emitted from the patch toward the observer's eye depends in part on its surface properties but also on the flow of light within the scene, the *light field* [4]. Our definition of light field is a simplification of the plenoptic function of Adelson & Bergen [5].

**Figure 2:**
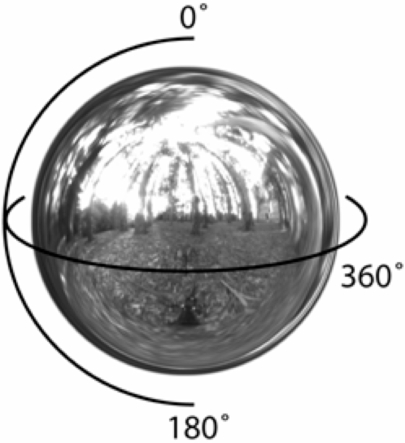
Lambertian bidirectional reflectance density function. The light field recorded at a point in a forest scene. The environment map for this illustration was obtained from http://www.debevec.org/Probes.

In this article we are concerned only with neutral (non-spectrally selective) lights and achromatic surfaces and, consequently, we can characterize a matte surface patch by its albedo and the light field as the intensity of light arriving at each point in the scene from every possible direction. In Figure 2 we plot the light field at one point in a forest scene as a spherical intensity map. To fully specify the light field, we would need to specify a similar spherical map at every location within the scene. We model matte surfaces as Lambertian: a small Lambertian surface patch absorbs light from all directions in a hemisphere centered on the patch, weighted by the cosine of the angle between the direction to the light source and the surface normal (See for details [6]).

Stable estimation of surface albedo (lightness) in complex, three-dimensional scenes requires that the visual system effectively discount this spatially-varying light field. Errors in judging either the spatial layout of the scene or the light field can potentially lead to failures in lightness constancy. We should note that we do not use the term “discounting of the illumination” to imply that observers have completely discounted the effect of variation in illumination or achieved perfect lightness constancy. We use it in a graded sense where we expect that the visual system attenuates but does not completely eliminate differences in illumination. Though we do not employ them here, this sense of the term is consistent with use of the Brunswick ratio or Thouless ratio as a graded measure of the visual system's success in discounting the illumination [7]–[8].

### Previous work

Based on earlier work [9]–[10], Doerschner and colleagues [6] characterized the problem of matte surface color perception in three-dimensional scenes mathematically. Given the evident complexity of the problem, it is surprising that observers do partially discount the illumination in scenes despite changes in surface location [11]–[23] and surface orientation [24]–[29]. One implication of these experimental results is that visual estimation of achromatic surface albedo in scenes depends on the three-dimensional interpretation of the scene including perceived depth.

The interpretation of a scene is typically the result of combining multiple cues to depth and shape [30] (See for review [31]) and the experiments cited above use a wide range of manipulation of depth cues. For convenience, we will refer to the set of available kinds of depth cues as the “mix” of depth cues. We next consider what mixes of depth cues are present in the experiments just considered.

Many of the articles cited compare monocular and binocular viewing of stimuli [24], [32] with the assumption that the visual system will interpret the change in viewing conditions as a change in scene organization. In some cases, single depth cues were altered in order to alter the observer's interpretation of the three-dimensional scene without changing the mix. Gilchrist [11], for example, manipulated an occlusion cue to alter the apparent depth of surfaces. Gogel and Mershon [13] altered the binocular disparity of a single test surface to effectively move it in depth while Mershon and Gogel [17] contrasted monocular and binocular viewing, altering the mix of available cues. Anderson and his colleagues [33]–[34] focused on monocular depth cues such as occlusion and transparency, and suggested that the surface segregation has a critical role in lightness perception. In these studies, the mix of depth cues varied with conditions and, moreover, binocular and pictorial depth cues were typically in conflict in one condition but not all.

In contrast, other studies altered real or rendered scenes without changing the available mix of depth cues [14]–[15], [27]–[29]. The depth information signaled by depth cues was always consistent across cues and consistent with the actual or simulated spatial layout of the real or rendered scene. In the experiments reported by Boyaci and colleagues [27]–[28], the experimenters also measured the perceived spatial layout of the scene to verify that the observer saw what the experimenter intended.

Viewed in detail then, the experiments summarized above vary any of three different factors across conditions (1) the mix of depth cues used, (2) whether the cues are in conflict or not, and (3) actual changes in location and orientation within real or rendered scenes.

### Goal of the present study

The results just reviewed demonstrate that, in many scenes, perceived depth affects perceived lightness. In this article we go beyond this result to examine whether different mixes of depth cues affect lightness. We presented observers with the same simulated scenes rendered by computer graphics methods but alter the mix and consistency of available depth cues. Pictorial cues were always present, and we systematically added or removed binocular disparity (BD) and motion parallax depth cues (MP).

One evident possibility is that the mix of depth cues present in a scene cues play little or no role. This is the hypothesis we test. This hypothesis is effectively assumed by several of the studies above which compare across conditions with different mixes of depth cues.

## Methods

### Observers

Seven undergraduate and graduate students participated in the experiment. All observers gave informed consent in writing. None of them were aware of the purpose of the experiment and all had normal eye acuity and normal stereo vision.

### Apparatus

Visual stimuli were generated and controlled by a computer (DELL Precision Workstation 530, Xeon 2.4 GHz, CPU, Nvidia Quadra 900XGL graphics) with the Open GL 1.0 graphics library. Stimuli were presented on a 21 inch CRT display (EIZO FlexScan T966; 1280×1024 pixel resolution, 38 cm width×30 cm height viewable area). We corrected the display for nonlinear gun responses using a standard gamma correction procedure.

Participants observed the display at 40 cm viewing distance (so that the display spanned 49.8 deg×39.8 deg in visual angle) with a chin-rest. Field-sequential shutter goggles (Stereographics CrystalEyes-3) were used for binocular stereo viewing. The visual image for left or right eye was presented alternatively at 100 Hz (50 Hz for each-eye image).

### Stimuli and conditions

**Figure 3:**
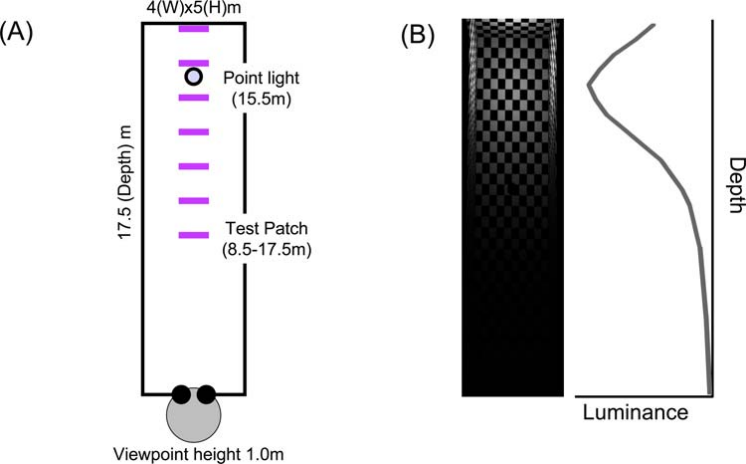
The rendered scene. *(A).* Top view of the rendered scene. The test patch (violet) could be at any of seven depths. *(B).* A plot of the actual intensity of light incident on the test patch as a function of depth, and a top view of rendered room.

**Figure 4:**
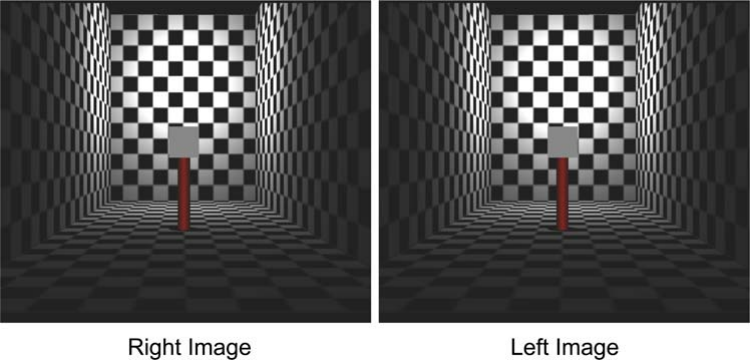
Stereo example of the scene. An example of the scene with pictorial cues and binocular disparity cues (for crossed fusion). The test patch is 6 meters from the back wall, 11.5 meters from the observer.

We created a virtual room (4 m width×5 m height×17.5 m depth) with checkerboard walls and floor (Figures 3, 4). In the rendered scene, each checker was 0.417 m×0.417 m. The observer's viewpoint was at the front-end of the room, centered on the scene, and 1.0 m height from the floor. Perspective (polar) projection was employed so that the rendered image always contained veridical pictorial depth cues such as linear perspective and texture gradient (Figure 4). A point light source was located at 15.5 m depth and 3.0 m height, and the rendered three-dimensional scene contained strong gradients of illumination in depth. The light source was not visible in the rendered scene presented to observers.

A test surface (0.5×0.5 m square patch) was put on a vertical pole (0.2 m diameter×1.0 m height) at 7 different positions in depth (8.5, 10, 11.5, 13, 14.5, 16, 17.5 m). Its retinal size was dependent on the distance from the observer (minimum 1.64×1.64 deg, maximum 3.37×3.37 deg). The pole supporting the test surface was rendered separately from the rest of the scene so as to avoid a possible local contrast cue where the pole joined the test surface. As a consequence, the shading on the pole is a pictorial cue that did not vary across depths and conditions and was not consistent with the lighting of the remainder of the scene. However, the resulting cue conflict should not affect comparisons across conditions in any important way because all conditions included the same pictorial cues. The test surface was independently illuminated at 4, 8, or 16 cd/m^2^ independent of position. Observers reported no difficulty in making lightness matches. The comparison surface did not appear self-luminous to the experimenters at any distance in any of the conditions in the experiment. The luminance of white checkers around the center of the back wall was more than 50 cd/m^2^. The actual luminance profile of a white surface (albedo *α* = 1) is shown in Figure 3b.

The scene was presented with or without binocular disparity (BD) cues to depth and with or without motion parallax (MP) cues to depth. Thus, there were 4 depth cue conditions, in total: Pictorial cues (PC) alone, PC+MP, PC+BD and PC+MP+BD. Binocular disparity was calculated by assuming that the observer's between-eye distance was 6 cm, and presented with a field-sequential shutter goggle. The participant observed all trials through the shutter goggle even when BD was not present. In the PC condition and the PC+MP conditions, the observer viewed the scene with both eyes but with binocular disparity depth cues set to zero disparity (bi-ocular viewing).

Motion parallax was simulated by moving the virtual viewpoint back and forth horizontally (1.0 m distance at 0.4 Hz). Thus, actually the room rotated on the display. We did not employ the head-yoked method [35]. However, the viewpoint motion in our display elicited a good depth impression as well. We refer to this cue as motion parallax.

The test surface was centered on a line perpendicular to the screen from the midpoint between the observer's eyes. The test surface always had zero disparity and remained almost stationary on the display even with motion parallax.

### Procedure

**Figure 5:**
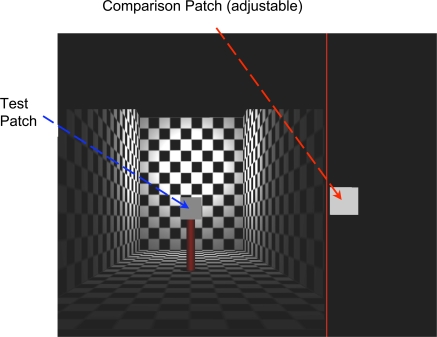
Test patch and comparison patch. The observer adjusted the luminance of the comparison patch until the perceived albedo of the patches was the same.

Observers were asked to adjust the luminance of a comparison surface so that the comparison surface matched the test surface in lightness. The test surface was placed at any of seven different depths in the scene. The comparison surface (1.91 deg×1.91 deg) was presented in the same location outside of the room scene and surrounded by a black background (Figure 5). Its luminance was adjusted by the participant using a mouse.

The duration of a trial was not limited and the participant observed the stimulus till he/she made a judgment. After the judgment, the next trial followed. Each participant performed 10 repetitions of combinations of 4 depth cue conditions, 3 levels of the test surface luminance, and 7 different depths in a random order (840 trials per observer). The stimulus array is shown in Figure 4 from the observer's viewpoint (pictorial cues and binocular disparity cue).

This research was approved by the Committee for Human-Subject Studies of the Toyohashi University of Technology.

## Results

**Figure 6:**
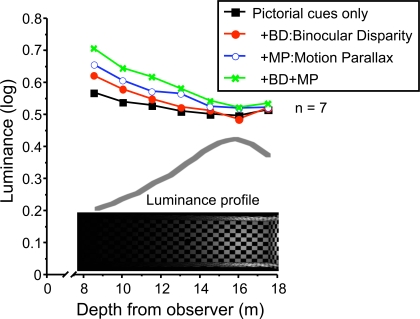
Discounting effects across depth cue conditions. The logarithm of luminance is plotted versus depth for each of the depth cue conditions, pictorial cues only (black solid square), pictorial cues and binocular disparity (red solid circle), pictorial cues and motion parallax (blue circle), pictorial cues, binocular disparity and motion parallax (green cross). The conditions of test surface luminance were merged here. The negative slopes indicate discounting of lightness. Luminance profile of the floor is superimposed. See text.

**Figure 7:**
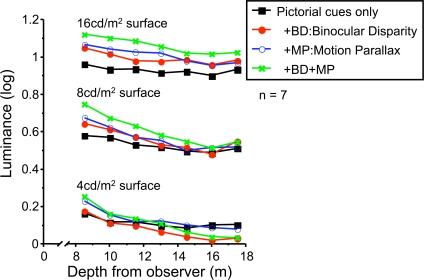
Discounting effects across depth cue conditions for test-surface luminance conditions. The logarithm of luminance for each surface-luminance condition (4, 8, 16 cd/m2) is plotted versus depth for each of the depth cue conditions like in Figure 6.

We plotted the logarithm to base 10 of luminance setting of the test surface against the distance between the surface and the observer (Figure 6, 7). We refer to these curves as “profiles.” A four-way repeated measures ANOVA (Luminance×Distance×Binocular disparity×Motion parallax) was conducted.

We next show that we can interpret the profiles are the relative perceived albedo of the test surface in logarithmic units as a function of depth. The values plotted on the vertical axis in Figure 6 are the mean luminance of the settings of the comparison surface *L_C_* when it is set to match a test surface of constant luminance, *L_T_*. We assume that (1) the comparison patch is perceived as a surface of adjustable albedo *αˆ* *_C_* under a constant but unknown illumination *Ê* *_C_* and (2) the test patch is perceived as a surface of albedo *αˆ* *_T_* under an illumination *Ê* *_T_* that varies with depth. The luminance settings then correspond to *Lˆ* *_T_* = *Ê* *_T_αˆ* *_T_* and *Lˆ* *_C_* = *Ê* *_C_* *αˆ* *_C_* and (3) if observers do follow instruction and set *αˆ* *_C_* = *αˆ* *_T_* (surface lightness match), We have
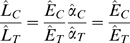
(1)and since *Lˆ* *_T_* is held constant by the experimenter and *Ê* *_C_* is assumed to be constant,
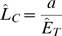
(2)where *a* is a constant and
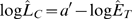
(3)where *a*′ is a constant. Moreover, since
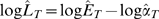
(4)and log*Lˆ* *_T_* is constant, we have
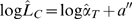
(5)where *a*″ is a constant. If the observer is following instructions then the profiles in Figure 6 can be interpreted as the observer's estimates of the logarithm of surface albedo of the test as a function of depth with an added, unknown constant (See Snyder, Doerschner & Maloney [20] for further discussion of this interpretation).

Consequently, with the assumptions just stated, we can interpret the profiles in Figure 6 and 7 as relative perceived test albedo in logarithmic units.

For all conditions, the perceived lightness decreased as the distance of the surface away from the observer increased (Main effect of distance, *F(6, 36) = 43.063, p<.0001*). That is, the test surface is perceived as lighter when it is near the observer. The largest difference induced by changes in the mix of depth cues occurs when the test patch is nearest to the observer (8.5 m). The increase in perceived lightness in going from pictorial cues alone to pictorial cues+MP+BD is about a 38% change in perceived lightness, with a surface perceived as albedo 0.5 at 16.0 m with only pictorial cues equivalent to surface of almost 0.7 with pictorial cues+MP+BD at 8.5 m. Since the scene had a strong gradient of illumination (darker in near depth to brighter in far), the result indicates observers partially discounted the illumination gradient in judging lightness, but much less than would be consistent with the actual luminance profile (Figure 3b). Adding binocular disparity and motion parallax led to significantly greater discounting (Interaction of binocular disparity and distance, *F(6, 36) = 5.791, p<.001*; Interaction of motion parallax and distance (*F(6, 36) = 13.13, p<.0001*). Thus, both depth cues enhanced discounting of three-dimensional illumination.

Illumination discounting was better with 4 and 8 cd/m^2^ test surfaces than with 16 cd/m^2^ test surface (Interaction of luminance and distance, *F(12, 72) = 5.207, p<.0001*). This might be because the brightest surface was perceived as self-luminous, independent of the room illumination. However, as noted above, none of the surfaces appeared self-luminous to the experimenters.

Even when observers had only pictorial depth cues and viewed the scene bi-ocularly (no MP, no BD), they partially but significantly discounted the illumination gradient in judging lightness. Adding either MP or BD led to greater discounting and both cues together significantly greater discounting. The effects of MP and BD were approximately additive across depths.

**Figure 8:**
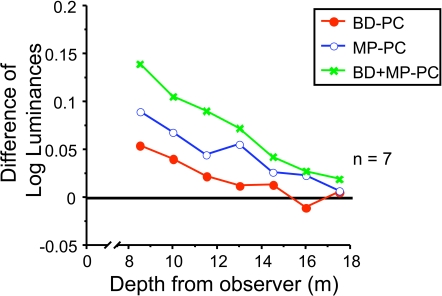
Depth Cue Effects without Pictorial Cues. Motion Parallax and Binocular Disparity. The effect of motion parallax alone, binocular disparity alone, and both motion parallax and binocularly disparity on luminance settings with the effect of pictorial cues subtracted.

In Figure 8, we plotted the differential effect of BD, MP, BD+MP depth cue conditions by subtracting the settings for the PC only condition. These plots show the additional discounting induced by MP, BD and MP+BD. Binocular disparity had greater influence at near distances than far.

**Figure 9:**
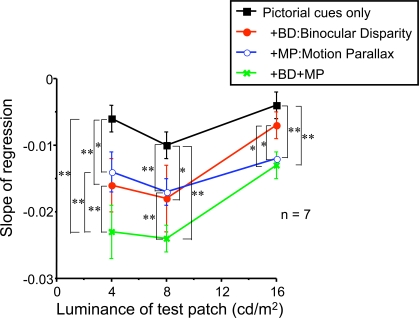
Depth cue effect analyzed by regression slope. The value of regression slope for each depth condition was plotted against test-patch luminance. Paired t-tests were conducted to test difference between conditions (* *p*<.05, **p<.01).

To quantify the discounting of illumination, we applied regression analysis to obtain the slope of perceived log lightness against depth. We plotted the value of slopes for each depth-cue condition against test-patch luminance (Figure 9). If this slope were steep (large negative value), it would indicate strong discounting. All values were significantly smaller than 0 (negative), indicating lightness discounting occurred for all conditions, even with only pictorial depth cues (single data t-test, *p<.05*).

A three-way repeated-measures ANOVA (Luminance×Binocular disparity×Motion parallax) for the slopes was conducted. The slope was steeper with 4 and 8 cd/m^2^ luminance surfaces than 16 cd/m^2^ surface (main effect of luminance *F(2, 12) = 10.96, p<.01*; post-hoc analysis Fisher's PLSD *p*<.05). This outcome is consistent with the previous analysis: discounting was weakened with the brightest test surface. The slope was steeper with BD or MP (main effect of BD *F(1, 6) = 18.15, p<.01*, main effect of MP *F(1, 6) = 29.34, p<.01*). This confirmed that binocular disparity and motion parallax led to increased discounting of the illumination gradient.

To test the significance of the differences between depth cue conditions, we conducted paired t-tests for each pair of slopes and report the results in Figure 9. For the 4 and 8 cd/m2 conditions, the results of the “all cues” condition (pictorial, BD and MP cues) was significantly different from those of the other conditions.

## Discussion

In three-dimensional scenes composed of neutral light sources and achromatic surfaces, the luminance of a matte surface depends on both its surface albedo and its location and orientation with respect to the light field across the scene. Stable estimation of albedo presupposes that the visual system takes into account the location and orientation of surfaces in such scenes. There is considerable experimental evidence that it does so [6], [11]–[29].

However, much research in lightness concerns pictorial scenes viewed bi-ocularly. The pictorial cues in the scene may signal variations in depth but binocular disparity cues signal, correctly, that the observer is looking at the flat surface of a picture. Logvinenko and colleagues [2] show that lightness perception in pictorial scenes can differ from lightness perception in actual 3D scenes viewed with binocular disparity. However, Kraft and colleagues [3] find little effect of switching from binocular viewing (correct disparity) to bi-ocular viewing (zero disparity). We examined whether lightness perception is affected by the mix of depth cues available and whether perceived lightness is different in bi-ocular and binocular viewing.

In the study reported here all cues were consistent with each another and with a single scene. Observers viewed a virtual room (4 m wide×5 m high×17.5 m deep). The walls and floors were covered with a checkerboard texture providing pictorial depth cues including linear perspective and texture gradient [30]. In four conditions, the room was presented with (1) pictorial cues only (PC), (2) pictorial cues and binocular disparity (BD), (3) pictorial cues and motion parallax (MP), or (4) pictorial cues together with both binocularly disparity and motion parallax depth cues (BD+MP).

In all conditions, observers were asked to adjust the luminance of a comparison surface to match the lightness of test surfaces placed at seven different depths (8.5–17.5 m) in the scene. We estimated lightness versus depth profiles in all four depth cue conditions. Even when observers had only pictorial depth cues, they partially but significantly discounted the illumination gradient in judging lightness. Adding either of the MP or BD cues led to significantly greater discounting and discounting was greatest with all cues combined.

A first implication of these results concerns experimental method. The perception of perceived surface albedo (lightness) with monocular viewing of scenes defined by pictorial cues alone is different from that in scenes with a richer mix of available depth cues that better approximate everyday viewing conditions. Observers showed a greater degree of discounting in binocularly viewed scenes with pictorial cues supplemented by motion parallax and binocular disparity. This outcome suggests that experiments that use only pictorial cues may lead to underestimates of the human ability to discount illumination in three-dimensional scenes.

Second, the differences in the perceived albedo (lightness) of constant-luminance stimuli increased markedly with distance from the observer. The largest difference was a 38% increase in perceived albedo and differences are both highly significant and patterned. Note, however, we tested surfaces that varied in depth from 8.5 m to 17.5 m. Had we confined attention to a narrower range we would have reported a smaller effect. However, this result suggests that experiments concerning lightness and color perception in three-dimensional scenes should include stimuli at a wide range of depths.

Last, we address the key issue raised by our results. Why should adding consistent depth cues to the mix of cues available in a scene alter perception of surface lightness and the inferred illumination gradient?

Could the effect we find be simply due to changes in perceived depth induced by changes in the mix of depth cues? If, for example, simultaneous contrast diminished with increasing separation between test and background, then changes in perceived depth due to changes in depth cue mix could lead to changes in perceived lightness. Snyder, Doerschner & Maloney [20] investigated lightness perception in binocularly-viewed rendered scenes with a strong gradient of illumination in depth (similar to the scenes employed here but with no variation in the mix of depth cues). They inserted specular spheres into the rendered scenes that signaled a gradient of light intensity increasing with depth (Exp. 2) or a gradient of light decreasing with depth (Exp. 3). The spheres floated in the air and were positioned away from the test surface and other landmarks; it is easy to verify that the spheres had little effect on perceived depth. However, the results (perceived lightness measured by asymmetric matching) for the two experiments were markedly different, an outcome inconsistent with the claim that perceived depth alone determined the effect.

When consistent cues are added to the mix of depth cues a scene, the resulting estimates of spatial organization are affected in two ways. The first is an increase in perceived depth in scenes viewed with addition of MP, BD or MP+BD that was evident to all observers. With pictorial cues alone, observers perceive a narrower range of depth as well as a shallower inferred illumination gradient. This effect is consistent with other reports of reduced lightness constancy with pictorial scenes [2]. However, it is not immediately obvious why observers should have reduced rather than exaggerated or veridical perception of both depth and illuminant gradients in scenes viewed with only pictorial cues. A decrease in perceived depth alone need not lead to a reduction in illuminant gradient if the light field scales with perceived depths in the scene. If anything, the inferred illumination gradient would be *steeper* if plotted versus foreshortened perceived depth with pictorial cues only.

The second way in which addition of consistent cues to the mix of depth cues in a scene could affect perceived spatial organization is best framed in terms of models statistical cue combination [31], [36]. We can at best make a qualitative argument on this point since we do not know how the visual system represents the light field or how depth information and depth cues contribute to estimating the flow of light in the scene.

Models of perception based on Bayesian decision theory [36] include the assumption that perception can be viewed in statistical terms. We can expect that observers have more accurate knowledge of both relative and absolute depth as we add depths cues of MP and BD. The light field in the current scene can be characterized as a particular setting of the parameters in a family of possible light fields. In estimating the lightness of achromatic surfaces, the parametric model needed would include a specification of the gradient of light in depth.

Bayesian decision theory provides a particular method for combining sensory information (“cues”) represented by a likelihood function with a prior probability distribution to arrive at estimates of parameters that describe the scene viewed. The prior on light fields represents information on the likely light fields that might be present in a scene. Mamassian & Landy [37], for example, investigated a prior on light fields corresponding to the well-known bias that observers have to interpret scenes with the assumption that the light source is above the scene. They were able to formulate the problem as a statistical estimation problem and estimate the prior (see [38] for discussion).

**Figure 10:**
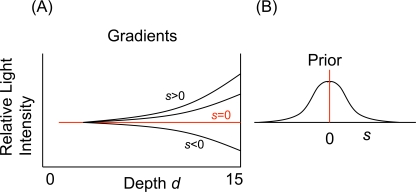
Prior for lightness perception. *(A)* Possible gradients of illumination indexed by a parameter *s*. *(B)* Prior distribution on the light field.

The relevant component of the prior in the context of the current article is the probability of different gradients of illumination in depth. Some examples of possible gradients are shown in Figure 10a indexed by a parameter *s*. Of course the visual system must simultaneously estimate other parameters describing the light field including parameters that characterize absolute light intensity and parameters that control segmentation of the scene into regions differing in illumination. Moreover, two or more parameters may be needed to characterize the gradient in depth. For illustrative purposes, we focus on the estimation of the gradient of light intensity with the assumption that a single parameter *s* is sufficient.

The observed effect of adding depth cues is consistent with the claim that added depth cues lead to a more reliable (lower variance) estimate of the light field and that cues to the light field available in the scene are combined with a prior distribution on the light field that favors uniform illumination in depth (s = 0, Figure 10b). Similar effects in the motion domain are reported in [39].

If the model we are proposing is correct, then the curves in Figure 6 are estimates of the gradient curves perturbed by measurement error. We do not know the probabilities that the visual system assigns to different gradients but we will assume that the *s* = 0 corresponds to the uniform (flat) gradient shown in red and that the prior distribution on *s* is Gaussian with mean 0 and variance 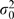. If we further assume that the variance of estimates of the gradient parameter decreases with added depth cues so that, with PC denoting pictorial cues, 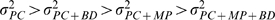 then Bayesian cue combination would result in estimates biased toward the prior *s* = 0 but with decreasing biases as the number of depth cues increases, the observed pattern.

Consequently we make the following conjectures that can be tested experimentally. (1) Changing the reliability of a single depth cue (without adding or subtracting depth cues) should also alter perceived lightness in scenes with strong gradients of illumination in depth and (2) in scenes where the actual gradient of illumination decreases with distance from the viewer, there should be a similar ‘flattening’ of the inferred gradient of illumination as depth cues are removed.

Bayesian approaches to perception are based on the assumption that the visual system makes use of prior probability distributions of possible scenes that could be innate or based on the statistics of past visual experience [40]–[41]. Adams, Graf & Ernst [42], for example, has shown that the light-from-above prior can be changed by interactive experience. Thus, if we observers are trained with one type of depth cue in a task not involving judgment of lightness but only judgment of location in depth, we might expect to see an effect on the perception of lightness in asymmetric lightness matching. Such experiments will serve to test Bayesian models of perception quantitatively and objectively.

There is now more than sufficient evidence to show that the visual system uses cues to three-dimensional spatial organization in estimating surface color and lightness and that studies restricted to flat, pictorial stimuli (‘the Mondrian singularity’ discussed by [43]) are not sufficient to characterize color and lightness perception. The current study carries the further implication that the mix of depth cues used in presenting three-dimensional scenes affects lightness perception and that the interaction of lightness and specific depth cues is rich and worthy of further study.

Our results suggest that there is a profound interaction between cues to location (including depth) and orientation on the one hand and perceived lightness on the other. This interaction raises the possibility that traditional accounts of lightness perception in pictorial stimuli will not readily generalize to three-dimensional scenes, a point made recently in a different context by Logvinenko, Petrini & Maloney [44].

Our results have implications for estimation of surface properties other than lightness. Recently, Motoyoshi and colleagues reported that image histogram moments (denoted IHM, e.g. the skewness of luminance distribution on images) account for the perception of lightness and glossiness of surfaces in nearly-flat scenes viewed binocularly [45]. In our results, however, the perception of lightness changed markedly as we varied binocular or motion parallax depth cues in scenes where there were pronounced differences in depth and surface orientation.

**Figure 11:**
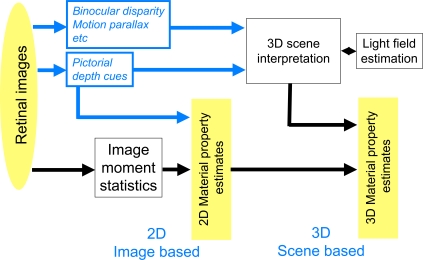
Two-stage model for material property perception The first stage (2D) makes use of pictorial cues including simple statistics such as image moments to estimate material properties including lightness. The second stage (3D) makes use of available depth cues to estimate scene layout and the light field and to further correct material property estimates based on the estimates of scene layout and light field. See text.

The differences in outcome in these two experiments (and many others) suggest a two stage model of processing of lightness and depth information (Figure 11; See also [43]).

We refer to the first stage as “2D processing”, where contrast/assimilation and image statistics control surface lightness and the scene is treated as effectively a flat image. In 2D processing, information such as IHM are available and can be used in estimating lightness and surface properties; 2D processing does not make use of the 3D interpretation of the scene and can also be described as “image-based” or “pictorial.” 2D processing is followed by a second stage (“3D processing”) wherein the 3D interpretation of the scene and the light field are both used to correct lightness perception for variations in the light field with changes in surface location and orientation in the scene. In scenes that are nearly-flat and uniformly illuminated, it is plausible that 2D processing based on IHM or other simple image statistics could account for observed variation in lightness perception (and perception of other material properties). In 3D scenes with non-uniform light fields, however, we would expect that IHM statistics alone would not adequately account for perceived lightness and material properties. The results presented here and in other studies discussed above demonstrate that IMH statistics alone cannot account for lightness perception. Similarly, Ho and colleagues find that perception of surface roughness also depends on surface depth and orientation and the light field [46]–[47]. If the Motoyoshi experiments were repeated with, for example, stimuli whose 3D structure were signaled by depth cues including motion parallax, we might learn that the visual system relies less on IHM when more powerful cues to gloss induced by object or ego-motion were available.

We conjecture that, in scenes with considerable variation in depth and surface orientation, subjects' estimates of surface gloss will vary in ways that cannot be readily explained in terms of IHM statistics as proposed by Motoyoshi et al. Second, to the extent that image moments are available earlier than a full scene interpretation in visual processing, we might expect that perception of surface lightness and other material properties will be less influenced by binocular disparity and motion parallax cues in 3D scenes that are briefly presented and masked.

One last point: we have presented the model above as if 2D processing preceded 3D processing, an assumption we consider plausible but by no means established. It may be that at least part of 2D processing associated with picture interpretation could occur late in visual processing. If so, one could as plausibly argue that an overall appearance is first determined by 3D scene interpretation and then modulated by 2D material cues such as IHM. An investigation of the time course of color and material perception could aid in better understanding how visual information about depth, orientation and light is combined in estimating lightness and other material properties.
